# Evolution of ‘whole institution’ approaches to improving health in tertiary education settings: a critical scoping review

**DOI:** 10.1080/02671522.2021.1961302

**Published:** 2021-08-25

**Authors:** Helen Sweeting, Hilary Thomson, Valerie Wells, Paul Flowers

**Affiliations:** a Institute of Health & Wellbeing, University of Glasgow, Glasgow, UK; b School of Psychological Sciences and Health, University of Strathclyde, Glasgow, UK

**Keywords:** University, college, settings approach, system, review

## Abstract

In recent decades, ‘whole school’ approaches to improving health have gained traction, based on settings-based health promotion understandings which view a setting, its actors and processes as an integrated ‘whole’ system with multiple intervention opportunities. Much less is known about ‘whole institution’ approaches to improving health in tertiary education settings. We conducted a scoping review to describe both empirical and non-empirical (e.g. websites) publications relating to ‘whole settings’, ‘complex systems’ and ‘participatory’/’action’ approaches to improving the health of students and staff within tertiary education settings. English-language publications were identified by searching five academic and four grey literature databases and via the reference lists of studies read for eligibility. We identified 101 publications with marked UK overrepresentation. Since the 1970s, publications have increased, spanning a gradual shift in focus from ‘aspirational’ to ‘conceptual’ to ‘evaluative’. Terminology is geographically siloed (e.g., ‘healthy university’ (UK), ‘healthy campus’ (USA)). Publications tend to focus on ‘health’ generally rather than specific health dimensions (e.g. diet). Policies, arguably crucial for cascading systemic change, were not the most frequently implemented intervention elements. We conclude that, despite the field’s evolution, key questions (e.g., insights into who needs to do what, with whom, where and when; or efficacy) remain unanswered.

## Introduction

This paper presents a scoping review of literature addressing ‘whole institution’ and ‘healthy setting’ approaches to improving health within tertiary education settings (i.e. post-secondary school settings, including universities and colleges offering both under-graduate/postgraduate degree courses and other academic or vocational qualifications). It provides a state of the art account of what has been published to date in this disparate area and charts the development and content of the available literature.

Many acknowledge that tertiary education settings may offer a critical site for health interventions. They represent a relatively bounded social system, large population numbers, high levels of need (exacerbated by the COVID-19 pandemic (Liu et al. 2020)) and stretched services (Broglia, Millings, and Barkham 2018; Gale and Thalitaya 2015; Haas et al. 2018; Holt and Powell 2017). Equally, within them, it may be possible to address the health of both students and staff simultaneously. The current review focuses on ‘whole institution’, population-wide, policy and/or environmental-level health interventions, which are likely to have greater reach and potential impact than interventions implemented at the individual level (e.g., supporting individuals with improving their mental health) alone (Capewell and Capewell 2017; Frieden 2010). Whole institution approaches have the potential to use a range of diverse mechanisms to effect change. These include micro (norms and social support), meso (culture and ethos) and macro-level mechanisms (the intersection of education and life chances), all of which can initiate and maintain health change beyond individuals and intra-individual mechanisms (e.g. cognition or affect) (Lewis et al. 2017; Mcleroy et al. 1988). In other words the whole institution, at every level, with diverse actors, in a range of interactions, becomes health promoting. Changes across a whole institution have the potential to take its social system to a tipping point where health-enabling processes and affordances are promoted throughout and mutually reinforce each other.

Paralleling the ‘whole institution’ approach, ‘settings-based’ health promotion has also emerged as a closely related, largely practitioner-led sister field. Therein health promoting activities have been characterised as ranging from the most conservative, ‘passive’ model (where the setting acts simply as a convenient space in which to deliver a ‘traditional’, individually-based intervention), to the most ambitious ‘comprehensive’ model, which ‘seeks to bring about direct and relatively significant changes in setting structure and culture within an assumption that individuals are relatively powerless to precipitate change to any significant level’ (Whitelaw et al. 2001, 344). Echoing the whole institution approach, the ‘healthy settings’ literature also emphasises the importance of understanding settings as ‘whole systems’ or ‘complex systems’, ‘with inputs, throughputs, outputs and impacts – characterised by integration, interconnectedness, interrelationships and interdependence between elements’ (Dooris 2006, 56). It also places a strong emphasis on principles of equity, partnership and stakeholder participation (Dooris 2013; Dooris et al. 2010, 2007; Shareck, Frohlich, and Poland 2013; Torp and Vinje 2014).

This approach has been developed by a number of tertiary education settings, with what have generally been described as ‘health promoting university’, ‘healthy university’ or ‘healthy campus’ initiatives in a range of different cultures and contexts (Suarez-Reyes and Van Den Broucke 2016). Although there have already been some reviews of this area, they have specifically focused on: theories/models used in relation to ‘healthy universities’ and ‘health promoting universities’ (Dooris, Wills, and Newton 2014); the implementation of ‘healthy universities’ and/or ‘health promoting universities’ (Ferreira, Brito, and Santos 2018; Reis et al. 2018; Suarez-Reyes and Van Den Broucke 2016) and university ’settings-based’ mental health interventions (Fernandez et al. 2016). None, so far as we are aware, have examined the extent and nature of the *broader* literature in this area, encompassing aspirational, theoretical and descriptive publications, as well as those presenting evaluations/trials relating to ‘whole settings’, ‘complex systems’ and ‘participatory’/‘action’ approaches to interventions aiming to improve the health, well-being and/or health-behaviours of students and/or staff within tertiary education settings. None have detailed the evolution of the current knowledge-base or the diverse terminology associated with the field. A search (March 2019, updated October 2020) within PROSPERO, an international database of prospectively registered systematic reviews focusing on health and social topics, did not identify any relevant reviews. We therefore undertook a scoping review.

Scoping reviews aim to map ‘the key concepts underpinning a research area and the main sources and types of evidence available’ (Mays, Roberts, and Popay 2001, 194). As such they differ from systematic reviews in focusing on broader topics and a range of study designs with little emphasis on quality; nor are they designed to perform detailed assessments or synthesis of findings data (Arksey and O’ Malley 2005). They are under-taken for a number of reasons, including, as here, to examine the extent, nature and range of (research) activity and to identify gaps in the existing literature (Hoffman et al. 2014).

The aim of our review was to determine what is included within the existing literature relating to ‘whole settings’, ‘complex systems’ and ‘participatory’/‘action’ approaches to interventions intended to improve the health, wellbeing and/or health behaviours of students and staff within tertiary education settings.

We addressed this via three research questions:

(1) What is the balance between different types of publication, particularly conceptual (aspirational/theoretical) versus empirical (descriptions/evaluations of such interventions)?
(2) What terminology has been used (as an indication of conceptual understandings and approaches – e.g., settings; systems; complexity; participatory/action approaches; healthy/health promoting university/campus)?
(3) Which population groups, health-related dimensions and activities have received most attention and are there clear gaps (as an indication of real-world actions)?

## Methods

### Search strategy

We aimed to identify *all* types of literature relevant to the review aim and research questions, including that reporting both non-empirical (e.g. theoretical papers and commentaries) and empirical work, and relevant charters and websites. We therefore searched five psychological, educational, social and health academic databases (Medline, PsychInfo, CINAHL, SCOPUS and ERIC) and four to identify grey literature (Directory of Open Access, Open Grey, e-theses online and Google Scholar) in July 2019 and again in September 2020. We also identified further key publications via the reference lists of studies which were read in full at the eligibility stage.

Our search strategy and inclusion criteria were based on the SPIDER (Sample, Phenomenon of Interest, Design, Evaluation, Research type) tool, appropriate for a broad range of research methods (Cooke, Smith, and Booth 2012), as follows:

- *Sample:* Those attending (i.e. students) or working in (i.e. staff) tertiary education institutions (i.e. post-secondary school education, including universities/‘higher education’ and colleges/‘further education’).
- *Phenomenon of interest:* Literature discussing, describing or relating to ‘whole settings’, ‘complex systems’ and ‘participatory’/‘action’ approaches and interventions aiming to improve health, wellbeing and/or health-behaviours within tertiary education settings (hereafter ‘whole’/‘healthy’ institution interventions).
- *Design:* All study designs.
- *Evaluation (outcome):* Mental health and wellbeing measures (e.g. measures of general mental health, wellbeing scales, measures of life satisfaction, happiness, resilience, self-esteem or quality of life); physical health and wellbeing; health risk behaviours (e.g. sexual health risk behaviour, smoking, excessive alcohol use, substance use, diet, exercise).
- *Research type:* All research types, together with all other non-research literature identified.
- *Other:* All English language academic and/or grey literature with no date restrictions.

Supplementary 1 shows the full inclusion and exclusion criteria and Supplementary 2 the final Medline search strategy (adapted as required for other databases); both were discussed and agreed by all authors.

### Selection of literature identified via database searches

**Figure 1:**
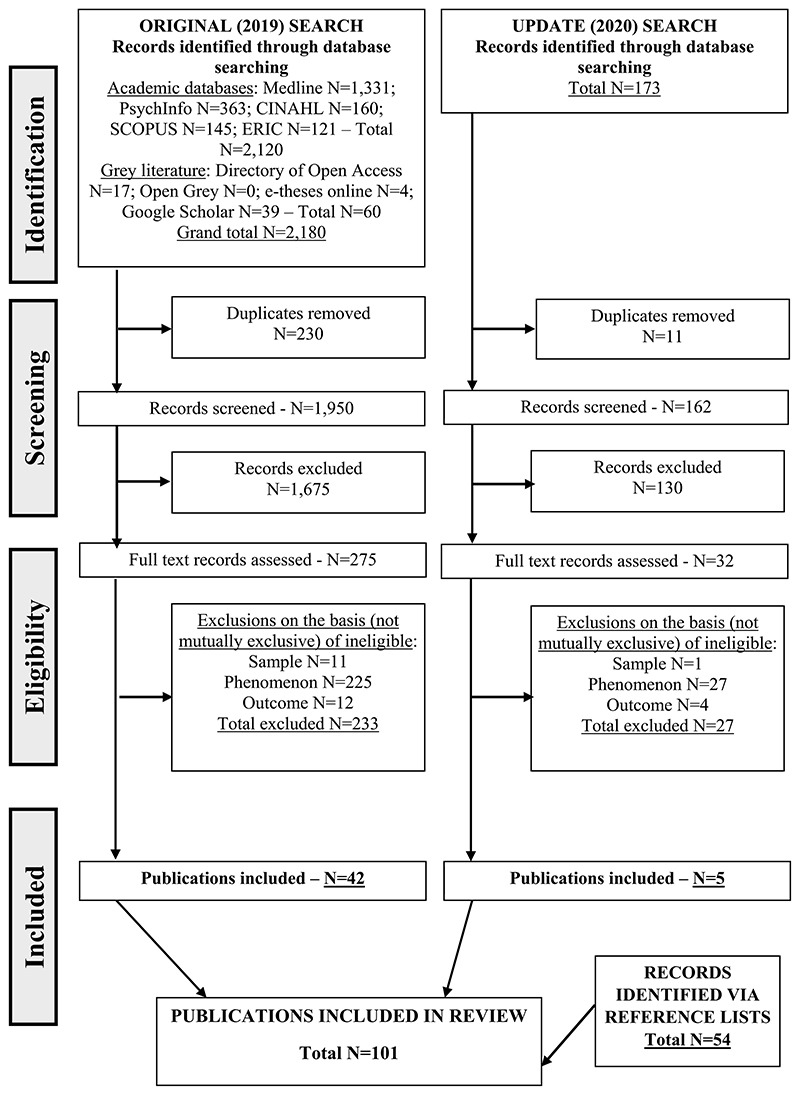
Flow chart of searches and exclusions.

Figure 1 shows a flow chart of all searches and exclusions from the two searches. Results from the original (2019) search were downloaded into Covidence (online software programme that supports the administrative management of systematic reviews) and assessed (by HS) against the inclusion and exclusion criteria. All those assessed as eligible for full-text assessment were read in full by HS, in randomly selected 10% blocks. The first random 10% were also read independently by PF, with discussion on the seven (of 27) where one or other was unsure of eligibility on the basis of ‘phenomenon of interest’ and subsequent tightening/clarification of the inclusion/exclusion criteria in respect of this. Decisions on the remaining 90% were made by HS. A similar process was conducted by HS in respect of the records identified in the 2020 update. We also included further publications (including some websites) identified via the reference lists of those read at the eligibility stage and assessed as meeting the inclusion criteria.

### Appraisal and coding

Since this was a scoping study aiming to provide an overview of all material reviewed, quality appraisal was not performed (Arksey and O’Malley 2005). The next stage was therefore to produce a coding frame to capture both basic publication details and also information on content. This involved an iterative process of reading, initial discussion (HS and PF), re-reading and trial coding before finalising the coding frame (Supplementary 3) which HS used to record information on:

- basic publication details (author; date; title; country of first author institution);
- funding and source if noted;
- publication format (e.g. journal article; book section; report; charter/declaration – see Supplementary 3.4 for full list);
- source (searches; reference lists);
- first author discipline (e.g. education; public health/health promotion – Supplementary 3.6);
- single or multiple authorship and, if so whether interdisciplinary;
- publication type (e.g. aspirational; observational studies; descriptions of the actions of a specific institution; evaluation – Table 1 shows all publication types and Supplementary 4 greater detail of criteria used to define type); if applicable, institution name and whether *any* (even somewhat vague) process, impact or outcome data were also coded in this section;
- use of words/terms representing the ‘whole’/‘healthy’ institution phenomenon in title/abstract (e.g. setting(s); whole system; healthy university/college – Supplementary 3.10);
- target groups or, if observational, the focus of data-gathering (e.g. students; staff – Supplementary 3.12);
- health dimensions referred to (e.g. attitudes/knowledge; smoking; wellbeing; ‘general health’ – Supplementary 3.13);
- for descriptions of the actions of a specific institution, evaluations or trials – who was involved in producing (e.g. students; senior/managerial staff; external organisations – Supplementary 3.14) and what activities were involved (e.g. physical environment; policies; promotions/marketing – Supplementary 3.15).

The coded data were entered into an SPSS datafile which aided data synthesis via the production of basic frequencies (Supplementary 3) and crosstabulations. Results are presented in the form of histograms, tables and narrative.

## Results

The original (2019) search identified 1,950 records after de-duplication, of which 275 were read in full and 42 were finally included; a similar process in respect of the 173 records identified in the 2020 update resulted in the final inclusion of five, resulting in a total of 47 publications identified via database searches. A further 54 publications (including some websites) identified via the reference lists of those read at the eligibility stage and assessed as meeting inclusion criteria were also included. The review was therefore based on 101 publications (identified via double asterisks in the reference list).

Our results begin by briefly describing the publications in terms of their format (journal article, book section, etc) and source (country, author disciplines and funding). The remainder of the results are structured according to our three research questions relating to the balance between different types of publication; the terminology that has been used; and the population groups, health-related dimensions and activities that have received most attention.

### What formats of publications were identified and what was their source?

#### Format

The 101 publications included 62 journal articles, 14 reports, 10 book sections, four websites, three journal editorials/commentaries, three dissertations/theses, three charters/declarations and two books (Supplementary 3.4). Identification of ‘academic’ publications (journal articles/editorials and dissertations/theses) was greater via the searches, and of both ‘non-academic’ (charters/declarations and websites) and ‘mixed’ publications (books/sections, reports) via reference lists (Supplementary 5).

#### Source

The publications originated from 13 countries: around half from the UK (46 publications, of which 22 included Dooris as an author, 16 of these as first author) and a quarter from the US (28 publications), with the remainder from Canada (9), Australia/New Zealand (6), other Europe (8) and Thailand/China (4). By far the most frequent first author discipline was public health/health promotion (48), followed by education (12), student (health) services (12), nursing (8), other health/medicine (5), psychiatry/psychology (4) and sociology/social work/social policy (3); 12 publications had organisational authors. Among the 89 with individual authors, 30 were single-authored, 36 by a multidisciplinary, and 23 a single-disciplinary team. Funding was noted by 39 publications, 24 naming health-related funders, five their institutions, three education-related funders and seven a range of other sources (Supplementary 3.1, 3.6, 3.2).

### Research Question 1: What is the balance between different types of publication, particularly conceptual versus empirical?

#### Numbers of each publication type

Table 1 details all 101 publications (author/s; date; country; title), categorised according to type and listed chronologically within each type. As it shows, a third (34) of the publications were *aspirational*, 16 of which were more general or broad *agenda-setting* (Table 1, refs 1 – 16) and 18 more specific guidelines or *‘road-maps’* (Table 1, refs 17–34). A further eight publications provided *general description of the ‘whole’/‘healthy’ institution concept* (e.g. the systems or characteristics of a healthy/health promoting university) (Table 1, refs 35–42). Although some of these aspirational/descriptive publications included brief examples of actions in one or more institutions, these were not their main focus. Six publications, some very brief, focused on *standards/measures* (Table 1, refs 43–48). Eighteen publications were *observational studies*. In seven of these, the ‘whole’/‘healthy’ institution concept was *peripheral*, used as a rationale or ‘hook’ for data-collection (e.g. student lifestyle surveys) or discussion of results (Table 1, refs 49–55). However, in 11 the concept was *central*, these publications presenting data specifically related to the ‘whole’/‘healthy’ institution (e.g. to provide recommendations for institutions’ (continued) provision of healthy settings) (Table 1, refs 56–66). A further 18 publications *described the actions* of 14 specific institutions (11 institutions each described in one publication; two institutions each described in two publications, so resulting in four publications; one described in three publications) with nine of these providing reflections on the process and four some (generally extremely brief) information on impact and/or outcome (Table 1, refs 67–84). Eleven publications reported *evaluations*, six of ‘whole’/‘healthy’ interventions in *one or a small number of institutions* (Table 1, refs 85–90) and five of policies/projects across *multiple institutions* (Table 1, refs 91–95), while another two reported on a single *randomised controlled trial* (Table 1, refs 96–97). Finally, four publications were *reviews* of ‘whole’/‘healthy’ institution interventions (Table 1, refs 98–101). Identification of publications focusing on standards/measurement, observational studies and evaluations in one or a small number of institutions was greater via the searches, and of aspirational publications, general descriptions and descriptions of the actions of single institutions via reference lists (Supplementary 5).

#### Evolution and geographical patterning of publication types – quantitative analysis

**Figure 2:**
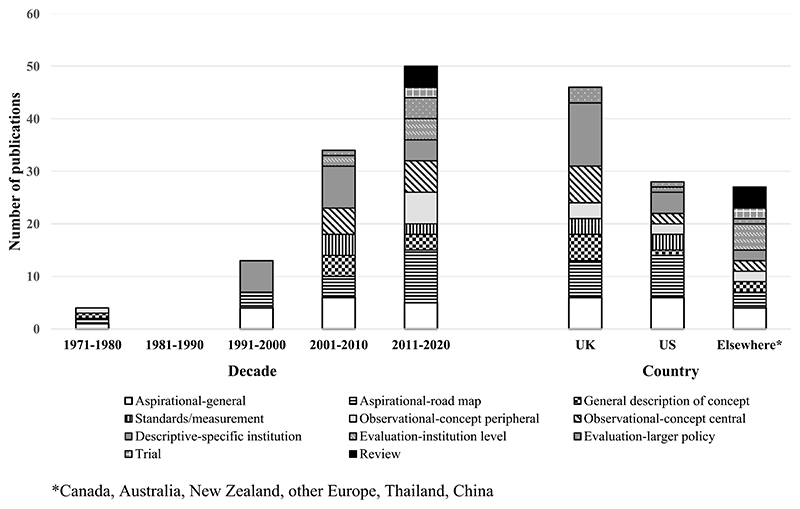
Number of each publication type according to publication decade and country.

Figure 2 shows the numbers of each publication type according to publication decade (representing evolution) and country (representing geographical patterning). It clearly highlights sharply growing interest in the field, from four publications during the 1970s, none in the 1980s and 13 in the 1990s, to 34 in the 2000s and 50 between 2011 and 2020. It also shows trends according to type. Thus, among aspirational publications, there was an increase between 1991 and 2020 in the proportion categorised as specific ‘road-map’, compared with general agenda-setting. While publications in the 1990s were only aspirational or described the actions of specific institutions, the 2000s saw the emergence of some more general descriptions of the characteristics of/concepts relating to a ‘whole’/‘healthy’ institution’ approach, interest in standards and measurement, observational studies with a central focus on the concept and a very small number of evaluations. Between 2011 and 2020, the number of evaluations increased, there was the first randomised controlled trial, the concept was being used by observational studies as a ‘hook’ for data-collection or discussion and the field had become sufficiently established to warrant (systematic) reviews.

Figure 2 also shows patterning by country of publication. A (far) larger number of publications from the UK described the characteristics of/concepts relating to a ‘whole’/‘healthy’ institution’ approach, the actions of specific institutions, observational studies with a central focus on the concept and larger policy evaluations. Around half the US publications were aspirational. Non-UK/US countries produced more evaluations of interventions in one or a small number of institutions, the only trial and all four reviews.

#### Evolution and geographical patterning of publication types – brief descriptive overview

The publication titles, provided in Table 1, give a flavour of the material identified. A detailed decade-by-decade description of this is available in Supplementary 6. In particular, it shows that although there were very few publications from the 1970s, all originated in the US and all provide evidence of ‘whole’/‘healthy’ institution thinking. Thus, they include mentions of ‘campus systems’ and an ‘ecosystem design process’ in a 1973 report from a task force set up ‘to explore applications of the community model as a means for resolving campus problems’ (Western Interstate Commission for Higher Education 1973); (alcohol) prevention strategies categorised as both specific (e.g. alcohol education) versus non-specific (not dealing directly with alcohol/drinking, e.g. providing alternatives such as physical activities, meditation or opportunities for creativity) and as individual versus environmental in the 1976 ‘Whole College Catalog About Drinking’ (Hewitt 1976); a category of mental health-related innovations described as ‘social engineering – attempts to alter the university environment’ in a 1974 paper presenting data gathered from university clinic directors (Winer et al. 1974, 282); and a 1979 conceptual paper describing the work of college mental health professionals within a general systems theory framework (Glazer 1979). However, development of the US national ‘road-map’ (‘Healthy Campus’) around 1990 was linked to assessment and broader national health objectives (Gordon 1995) rather than, as in the UK and else-where, being informed by the whole-system settings approach to health promotion (Tsouros, Dowding, and Dooris 1998). Within the UK, not only the research but also conceptual thinking and practical developments related to the approach have been largely driven by one individual. Publications from the UK are also marked by an interest in applying the approach to Further Education colleges in the first decade of the new millennium, which disappeared after 2010. Numbers of publications originating from countries outside the US and UK have been relatively small, but, strikingly, include the first evaluation, of a project conducted in China, which began in 1997 and aimed to ‘create health promoting universities within the framework of the Ottawa Charter’ (Xiangyang et al. 2003, 107), the only formal trial (Reavley et al. 2014a; b) and (systematic) reviews (Fernandez et al. 2016; Ferreira, brito, and Santos 2018; Reis et al. 2018; Suarez-Reyes and Van Den Broucke 2016).

### Research Question 2: What terminology has been used?

#### Use of conceptual terms

In order to capture the balance, evolution and geographical patterning of conceptual understandings and approaches within the literature. we coded for use of any words/terms representing the ‘whole’/‘healthy’ institution phenomenon in titles and/or abstracts (e.g., settings, systems, complexity, participatory/action approaches, healthy/health promoting university/campus). The word ‘setting(s)’ was included most frequently, occurring in the title/abstract of around a third (N = 36) of the publications (Supplementary 3.10). ‘Whole system’ occurred in 13 publications, while ‘whole’/‘holis-tic’ and ‘system(s)’/‘systemic’ (not ‘whole system’) each occurred in 11. ‘Participatory action/process/research’ and ‘complex’/‘complexity’ (not ‘complex system’) each occurred in six publications and ‘complex system(s)’ in one; no publication included the term ‘complex adaptive system(s)’. Any other broad term suggesting ‘whole’ (e.g. ‘campus ecology’) occurred in 54 publications (Supplementary 3.11 lists all such terms). In addition, around a third of the publications mentioned ‘healthy(ier) university/college’ (N = 35) and/or ‘health promoting university/college’ (N = 33) and just over one-in-ten ‘healthy(ier) campus’ (N = 13).

#### Evolution and geographical patterning of conceptual terms

As Table 2 shows, these terms were also patterned by both decade (evolution) and country (geographical patterning of conceptual understandings). Thus, there was evidence of the emergence/evolution of ‘settings’, ‘systems’-related and ‘participatory’ terms, which largely began in the 2000s as did ‘healthy(ier)’ and ‘health promoting’ university/college and, more clearly in the 2010s, ‘healthy campus’. In contrast, the proportion of publication titles/abstracts using other broad terms remained fairly stable over time. In respect of country differences, the term ‘setting(s)’ was used by almost no titles/abstracts from the US, but by approaching half those from the UK and over half from elsewhere. Similarly ‘systems’-related words were barely used in titles/abstracts from the US or elsewhere, but in over a quarter of the UK ones, however no UK title/abstract used ‘participatory’ terms. While almost no US title/abstract used ‘healthy(ier)’ and ‘health promoting’ university/college, these were both (particularly ‘healthy(ier)’) commonly used in the UK and (particularly ‘health promoting’) elsewhere. In contrast, around a third of the titles/abstracts from the US included ‘healthy(ier) campus’, compared with only a small number from elsewhere and none from the UK.

#### Linkages between conceptual terms

Finally, these conceptual terms were not used in a mutually exclusive way, and further analyses (Supplementary 7) suggested linkages between particular concepts: ‘systems’-related words were used in association with ‘setting(s)’ but not ‘participatory’; ‘setting(s)’ and ‘systems’-related words with ‘healthy(ier)’ and ‘health promoting’ universities/colleges, but not with ‘healthy campus’; and neither ‘participatory’ nor ‘healthy campus’ were used with ‘healthy(ier)’ universities/colleges.

### Research Question 3: Which population groups, health-related dimensions and activities have received most attention?

#### Population groups

As Table 3 shows, the vast majority (N = 87 of the 101) of publications included a focus on students, almost three-quarters (N = 73) on staff and a quarter (N = 28) on organisations or individuals in the wider (external) community as either benefitting from being part of an institution taking a ‘whole’/‘healthy’ approach to health and wellbeing or as the focus of data collection.

#### Health-related dimensions

Table 3 also shows that most publications focused on ‘health’ generally rather than specific health dimensions. Of those that did specify, lower-level mental health and wellbeing issues were most commonly mentioned, followed by nutrition, smoking, alcohol, formal psychiatric illness, physical activity, sexual behaviour and drugs. Only small numbers included health service use, health attitudes/knowledge, aggression or physical health.

#### Activities

As noted above, descriptions were available in respect of the specific actions of 14 different institutions; in addition, six publications reported institution-based evaluations occurring in four further institutions and two reported on the same randomised controlled trial. Information on who was involved in producing the intervention and what activities were involved were therefore available for 19 different institutions. In cases where information in respect of a single institution differed slightly between publications (e.g. (Dooris 1998, 2001, 2002; Mendenhall et al. 2011, 2014, 2008)), a group/activity mentioned in any version ‘counted’, since some versions provided more detail, or were written later, when the intervention may have been more developed. As Table 4 shows, in all or almost all cases where details were provided, students, senior/managerial and/or health centre staff were in some way involved in producing the intervention, around half involved teaching, catering/physical activity and health promotion staff and/or external organisations, and a small number had a specific co-ordinator. In addition, four of the 19 were unclear but comments suggested wide involvement and five provided no clear description of who was involved. Table 4 also shows that by far the most frequent activities were promotions/marketing and health education. Policies, surveys/data-gathering, changes to health services and staff wellbeing support were described in around two-thirds and changes in the physical environment, catering, physical activity provision and learning, and student projects/committees in around half. Smaller numbers described partnerships/relationship-building, staff training, student peer-to-peer activities, a dedicated website and changes to procurement. In addition, three were unclear but comments suggested wide-ranging activities and five provided no clear comments on activities.

## Discussion

This scoping review, based on a search of five academic and four grey literature databases with no date restrictions and additional publications identified via the reference lists of studies read at the eligibility stage, identified 101 items, published between 1973 and 2020. It represents a key contribution by combining a general overview of the field, giving a flavour of the literature overall, with more specific details and analyses to address a series of research questions.

Our review aimed to determine what is included within the literature relating to ‘whole settings’, ‘complex systems’ and ‘participatory’/‘action’ approaches to interventions intended to improve the health, wellbeing and/or health behaviours of students and staff within tertiary education settings. A simple answer is that it is highly diverse, international and interdisciplinary in nature. Our review has also demonstrated that consideration of issues relating to ‘whole’/‘healthy’ universities and colleges has a long history, stretching back to the 1970s, earlier than the 1990s generally suggested in publications describing ‘health promoting’/‘healthy’ universities, and that it has evolved over time.

Our first research question related to the balance between publications with a conceptual focus (aspirational/theoretical) versus those with an empirical focus (intervention descriptions and/or evaluations) within this literature. We have shown that the balance has changed over time and that the literature has evolved from being aspirational and theoretical to reporting, to a greater or lesser extent, how the approach has been implemented and evaluated. In this way there is a sense of the field maturing over time, from ideas to real-world actions. However, aspirational publications continue to be published and the number of published evaluations or trials in individual or a small number of institutions remains relatively small, with the first, conducted in Beijing and published in 2003 (Xiangyang et al. 2003), arguably still the most comprehensive, and very few studies including clear before-after outcome comparisons. Thus, although the field has evolved, there is a sense of uneven progress internationally and some lost momentum.

Our second research question focused on terminology used as an indication of conceptual understandings and approaches. We addressed this via an examination of words within titles and abstracts. ‘Setting(s)’, ‘healthy university/college’ and ‘health promoting’ were each used in almost a third of the publications, ‘system’ or related terms and ‘healthy campus’ in rather fewer and ‘participatory’ and ‘complex’ in only a handful. There were no mentions of ‘complex adaptive system’, which was a term we had thought we might find since it describes what many of the ‘whole’/‘healthy’ institution interventions were aiming for (a dynamic network of interacting agents, adapting as required and working via feedback loops) and has been used extensively in association with health promoting schools (Keshavarz et al. 2010). It is also of interest that there seemed to be little cross-referencing with the literature on health promoting schools and whole school approaches, which also originated from the concepts of settings-based approaches to health and systems thinking but has a more established research base (Langford et al. 2015; Thomas and Aggleton 2016). One difference may be clearer long-term World Health Organisation support for the approach within schools (World Health Organisation 2018) than tertiary education settings (Orme and Dooris 2010).

These conceptual title/abstract terms were patterned by geography, with UK-based publications using both ‘settings’ and ‘systems’, those from elsewhere only ‘settings’ and those from the US neither. Equally, as others have noted (Dooris, Powell, and Farrier 2020; International Conference on Health Promoting Universities and Colleges/VII International Congress 2015), ‘healthy university’ was used only in the UK and ‘healthy campus’ in the US, while ‘health-promoting’ was favoured elsewhere. The finding that ‘setting(s)’ and ‘systems’-related words were used with ‘healthy(ier)’ and ‘health promoting’ universities/colleges but not with ‘healthy campus’ underlines the origins of the ‘Healthy(ier)’ and ‘Health Promoting’ universities/colleges movements from settings-based health promotion (World Health Organisation 1986), while ‘Healthy Campus’ began in response to national US health objectives. However, the (geographically) siloed nature of the literature, as well as its interdisciplinarity, may also have reduced the potential for building international momentum or accumulating a clear and transferrable evidence-base. Somewhat relatedly, the scoping review identified that almost all UK ‘whole’/‘healthy’ universities work, represented by aspirational (Doherty, Cawood, and Dooris 2011; Dooris 2010; Dooris et al. 2010; Dooris and Doherty 2008; Orme and Dooris 2010; Tsouros, Dowding, and Dooris 1998), description (in relation to both concept (Doherty and Dooris 2006; Dooris 2006; Dooris, Doherty, and Orme 2017; Dooris, Wills, and Newton 2014) and institutional actions (Dooris 1998, 2001, 2002)), observational (Dooris and Doherty 2009, 2010a; b; Dooris, Powell, and Farrier 2020; Holt et al. 2015; Newton, Dooris, and Wills 2016) and evaluation (Dooris et al. 2018, 2019; Dooris and Powell 2012) publications, has been led by one author (Dooris), supported by his institution which hosts the national road-map (‘Healthy Universities’) website (University of Central Lancashire & Manchester Metropolitan University 2020), in contrast to the equivalent US and Canadian websites, which are hosted by national/regional bodies (American College Health Association 2018; Canadian Mental Health Association 2020). While this represents extensive and impactful achievements, it could be argued as Dooris has himself (Dooris, Wills, and Newton 2014) that it also corresponds to just one perspective, when it is increasingly recognised that complex problems are likely to benefit from an evidence base built on the work of multiple researchers with potentially different perspectives (Bennett and Gadlin 2012).

Our third research question related to which dimensions of health, wellbeing and/or health-behaviours have received the most attention within this literature. In fact, the majority of publications simply refer to ‘health’, signifying the ‘whole’/‘healthy’ institution approach. While ‘health’ might be appropriate for general agenda-setting aspirational publications and reflects the interrelated nature of mental and physical health, it could be argued that designing exactly what a ‘whole’/‘healthy institution might look like requires consideration of more specific health dimensions in order to identify mechanisms and perhaps prioritise potentially distinct activities associated with particular health outcomes identified as important by the target population group(s). More specific health dimensions identified in the literature (mental wellbeing, nutrition, smoking, alcohol) demonstrate, as noted by others (Suarez-Reyes, Serrano, and Van Den Broucke 2019; Suarez-Reyes and Van Den Broucke 2016), that interventions of this type focus on health issues which are common among young people. Students and senior/managerial staff were most frequently described as involved in producing the intervention, fulfiling the requirements of a process that needs both bottom-up and top-down activities (Dooris 2001, 2002). Others have suggested that institutions are most likely to choose ‘whole’/‘healthy’ actions that are closest to their mission (e.g. health education; support for health promotion research; changes to teaching/assessment) (Fernandez et al. 2016; Suarez-Reyes and Van Den Broucke 2016). Such activities may also help an institution to ‘tick the box’ in respect of addressing the quality of student experience, which is increasingly required as part of both internal and external quality reviews (Shah, Nair, and Richardson 2017). The activities we identified as most often described (promotions/marketing; health education) are likely to be easier to implement than broader high-level policies (e.g. a corporate policy on health (Dooris 2001); rules around permissible smoking locations; administrative approvals and provision of resources for various social/physical activities (Mendenhall et al. 2011)), which are regarded as crucial for significant and sustained systemic change (International Conference on Health Promoting Universities and Colleges/VII International Congress 2015; Second International Conference for Health Promoting Universities 2006; Tsouros, Dowding, and Dooris 1998), but were described in only two-thirds of institutions.

### Limitations

Like all reviews, ours was bound by decisions relating to inclusion/exclusion categories and choice of search terms. While the latter aimed to be broad, one inclusion criterion was English language, thus excluding some literature, including some (both theoretical and empirical) from Latin America, which has been incorporated in other reviews (Ferreira, Brito, and Santos 2018; Reis et al. 2018; Suarez-Reyes and Van Den Broucke 2016).

Related to this, our SPIDER tool (Cooke, Smith, and Booth 2012) ‘Phenomenon of Interest’ was fuzzy and therefore subjective in a similar way to definitions used by others working in this area, who have referred to interventions ‘at institutional level’ and including ‘the whole community’ (Dooris, Wills, and Newton 2014; Suarez-Reyes, Serrano, and Van Den Broucke 2019). We frequently asked ourselves, particularly in the early stages of selection, how ‘whole’ is ‘whole’, since the variety of publications meant it was not possible to set criteria which could be universally applied. Initial over-inclusiveness (e.g. a smoke-free campus initiative; papers with a brief nod towards the idea that student health issues require more than just individual-level/health centre-based responses) was rejected, but some decisions were only made after several re-readings, and other reviewers might have drawn the line differently.

Similarly, our categorisation of publication type (as aspirational, observational studies; descriptions of the actions of a specific institution; evaluation; etc), while far more nuanced than, for example, ‘theoretical’/’intervention’ (Reis et al. 2018; Suarez-Reyes and Van Den Broucke 2016) meant that judgement was required in respect of how some should be classified (e.g., those which were broad enough to ‘fit’ more than one category (Newton 2014) or borderline between categories (Dooris and Doherty 2010b)). Again, others might have made some different decisions. (Note that while mindful of the benefits of more joint screening and decision-making, (Arksey and O’Malley 2005), this was not possible because the second reviewer was diverted to coronavirus-related work.) However, it is unlikely that others would have disagreed in respect of most publications and categories, so the broad mapping, which is the purpose of a scoping review, would likely be largely replicated. An additional issue is that much of the final coding frame, including not only publication type but also categories in respect of what groups, health-related dimensions, varieties of leadership and activities have received most attention, emerged from reading and re-reading the papers, and in that sense was inductive. Although this means our categorisations cannot be directly mapped onto those set out in aspirational charters (International Conference on Health Promoting Universities and Colleges/VII International Congress 2015; Second International Conference for Health Promoting Universities 2006) or the findings of others (Dooris and Doherty 2010b; Suarez-Reyes, Serrano, and Van Den Broucke 2019), they are, unsurprisingly, very similar.

A further issue in respect of target groups, health-related dimensions, varieties of leadership and activities, is that our coding of any intervention was, of course, based on information provided by the authors. This itself may have been incomplete, or based on descriptions drafted early on within the intervention development process. Where multiple versions of intervention descriptions presented slightly different accounts across papers, something mentioned in any version ‘counted’, but some descriptions were unclear, for example, in respect of whether activities had been implemented or just planned.

### Implications

This scoping review and its detailed supplementary materials should form a comprehensive resource for those wanting an overview of the literature relating to ‘whole’/‘healthy’ institution approaches to improving the health of students and staff within tertiary education settings. In addition, it has particular implications in respect of both what is required in order to progress the field and the methods used for scoping studies such as ours.

The balance of publication types identified suggests strongly that what is now required is evaluations of ‘whole’/‘healthy’ interventions in tertiary education settings. There are very significant challenges in both implementing (Dooris 2002) and evaluating such interventions (Budgen et al. 2011; Doherty, Cawood, and Dooris 2011; Dooris 2006; Suarez-Reyes and Van Den Broucke 2016; Whitehead 2004), since in order to be effective, they require understandings of the unique and shared (multi-level) determinants of a range of selected health dimensions and behaviours; co-production to decide the priorities on which to focus; and harmonised modification of the determinants that relate to the institutional setting (policies; power; interactions; resources; curriculum). In recent years, much has been written about the importance of intervention development (O’Cathain et al. 2019) and providing transparent accounts of intervention content and associated mechanisms (Craig et al. 2008). The large population numbers, high levels of need and stretched services highlight a requirement for increased understanding of the efficacy and mechanisms of ‘whole’/‘healthy’ approaches within tertiary education settings based on these ideas. The geographically siloed nature of the literature identified in our review suggests that progress towards this goal might be most efficiently achieved via more international as well as more interdisciplinary collaboration, to build bridges in concepts, approaches and terminology to support the programmatic development of a larger evidence base.

One of our research questions included the possibility of gaps within this literature in respect of population groups, health-related dimensions or activities. One very clear gap, within the UK, is the Further Education sector. UK Further Education takes place in colleges rather than universities, generally equips students for further learning (including university-based Higher Education) or employment and includes students from more disadvantaged groups. Our review suggests that despite its relatively early emergence, activity relating to ‘healthy colleges’ (i.e. occurring within Further Education) ceased around 2010. While it is possible that work is continuing but undocumented, a national ‘Healthy FE’ website referred to in one road-map publication (Marshall and Stylianou 2010) has ceased to exist. This is despite the fact that students within Further Education (and equivalent institutions elsewhere (Mendenhall et al. 2008)) may be particularly vulnerable (Warwick et al. 2006) and so highly likely to benefit from investments in health.

Finally, and from a methodological standpoint, it is unsurprising that ‘academic’ publications were more likely to be identified by the searches and ‘non-academic’ and ‘mixed’ publications via reference lists. However, the fact that this also meant the searches identified different publication types (e.g. fewer aspirational publications) has methodological implications, underlining the importance of not relying on searches, even those designed to identify grey literature, in a scoping study such as ours.

## Conclusions

‘Whole institution’ approaches to improving health within tertiary education settings have evolved from a handful of agenda-setting aspirational publications in the 1970s to road-map websites and charters and growing international recognition. However, progress towards a solid and significant research evidence base has been relatively slow. The challenges are enormous, both for institutions aiming to fully, rather than tokenistically implement such interventions and for researchers aiming to evaluate them within a funding and evidence context that is skewed towards trials, short-term outcomes and simple linear models of cause and effect. Our review would suggest there is a need to build on existing leadership and expertise, and invest resources in the development of a robust and detailed programme theory (Rogers 2008) and evaluability assessment rather than large scale trials or natural experiments at this point in time, in order to further develop this field.

## Figures and Tables

**Table 1:** Publication details (author, date, country and title) categorised according to type.

	Author(s)	Date	Country	Title
Aspirational – general, broad agenda-setting: no/little data (may include supporting refs, very brief examples, but publication not focused on these)				
1	Western Interstate Commission for Higher Education	1973	US	The Ecosystem Model: Designing campus environments
2	Patrick et al.	1992	US	Health issues for college students
3	Gordon	1995	US	College health in the national blueprint for a Healthy Campus
4	Jackson and Weinstein	1997	US	The importance of healthy communities of higher education
5	Tsouros	1998	UK	From the healthy city to the healthy university: project development and networking
6	James	2003	UK	A Health Promoting College for 16-19 year old learners
7	Fabiano and Swinford	2003	US	Serving higher education communities with health promotion
8	Second International Conference for Health Promoting Universities	2006	Canada	The Edmonton Charter for Health Promoting Universities and Institutions of Higher Education
9	Dooris and Doherty	2008	UK	English Healthy Universities Network: Framework for action
10	Dooris	2010	UK	Healthy Universities: Introduction and model
11	Orme and Dooris	2008	UK	Integrating health and sustainability: the higher education sector as a timely catalyst
12	Doherty et al.	2011	UK	Applying the whole-system settings approach to food within universities
13	International Conference on Health Promoting Universities and Colleges/VII International Congress	2015	Canada	Okanagan Charter: An international charter for health promoting universities and colleges
14	Lederer and Oswalt	2017	US	The value of college health promotion: A critical population and setting for improving the public’s health
15	Taylor et al.	2017	Australia	Creating healthier graduates, campuses and communities: why Australia needs to invest in health promoting universities
16	Came and Tudor	2020	New Zealand	The whole and inclusive university: a critical review of health promoting universities from Aotearoa New Zealand
Aspirational – specific guidelines/’road-map’: no/little data (may include supporting refs, very brief examples, but publication not focused on these)				
17	Hewitt	1976	US	The Whole College Catalogue About Drinking: A Guide to Alcohol Abuse Prevention
18	O’Donnell and Gray	1993	UK	The Health Promoting College
19	Tsouros et al.	1998	UK	Strategic framework for the Health Promoting Universities project
20	Tsouros and Dowding	1998	UK	A framework for action by a European Network of Health Promoting Universities
21	National Association of Student Personnel Administrators	2004	US	Leadership for a healthy campus: An ecological approach for student success
22	Filkowski	2008	US	Leadership for campus mental wellness
23	Dooris et al.	2010	UK	Healthy Universities: Concept, model and framework for applying the healthy settings approach within higher education in England
24	Marshall and Stylianou	2010	UK	A Practical Guide to becoming a Healthy College
25	Drum and Denmark	2012	US	Campus suicide prevention: bridging paradigms and forging partnerships
26	Canadian Mental Health Association	2013	Canada	Post-secondary student mental health: Guide to a systemic approach
27	Partnership for a Healthier America	2014	US	Healthier Campus Initiative (website)
28	University of Central Lancashire	2015	UK	UCLan Healthy University Action Plan 2015-18
29	American College Health Association	2018	US	Healthy Campus (website)
30	National Institute on Alcohol Abuse and Alcoholism	2019	US	Planning alcohol interventions using NIAAA’s CollegeAIM Alcohol Intervention Matrix
31	American College Health Association	2020	US	The Healthy Campus framework
32	Canadian Mental Health Association	2020	Canada	Healthy Minds, Healthy Campuses (website)
33	Innstrand and Christensen	2020	Norway	Healthy Universities. The development and implementation of a holistic health promotion intervention programme especially adapted for staff working in the higher educational sector: the ARK study
34	University of Central Lancashire & Manchester Metropolitan University	2020	UK	Healthy Universities (website)
General description of the ‘whole’/’healthy’ institution concept: what it means/is, what characterises it - no data (may include case study/ies but publication not focused on these)				
35	Glazer	1979	US	General systems theory and college mental health professionals
36	Whitehead	2004	New Zealand	The health promoting university (HPU): the role and function of nursing
37	Doherty and Dooris	2006	UK	The healthy settings approach: the growing interest within colleges and universities
38	Dooris	2006	UK	Healthy settings: challenges to generating evidence of effectiveness
39	Warwick et al.	2008	UK	Healthy and health promoting colleges - identifying an evidence base
40	Dooris et al.	2014	UK	Theorising healthy settings: a critical discussion with reference to Healthy Universities
41	Racher et al.	2014	Canada	Taking the right action in the right way: a comparison of frameworks for assessing the health and quality of life of a postsecondary student campus community
42	Dooris et al.	2017	UK	The application of salutogenesis in universities
Standards/measurement (development)				
43	Zimmer et al.	2003	US	A scope-of-practice survey leading to the development of standards of practice for health promotion in higher education
44	birch	2006	UK	Kirklees Healthy College Standard
45	Ahern	2007	UK	Stockport Healthy College Standard: an audit tool for Every Child Matters
46	Balding	2007	UK	Support for healthy colleges
47	Sowers et al.	2017	US	Survey development to assess college students’ perceptions of the campus environment
48	Horacek et al.	2019	US	Development and validation of the Policies, Opportunities, Initiatives and Notable Topics (POINTS) Audit for Campuses and Worksites
Observational study – whole/healthy institution concept is peripheral: used as rationale or hook for data collection or ‘baseline’ in terms of not following specific actions				
49	Winer et al.	1974	US	Innovations at university mental health services
50	Stock et al.	2014	Denmark	Student estimations of peer alcohol consumption: links between the Social Norms Approach and the Health Promoting University concept
51	Holt and Powell	2017	UK	Healthy Universities: a guiding framework for universities to examine the distinctive health needs of its own student population
52	Murphy	2017	Ireland	Responding to the needs of students with mental health difficulties in higher education: An Irish perspective
53	Haas et al.	2018	UK	Changes in student physical health behaviour: an opportunity to turn the concept of a Healthy University into a reality
54	Hartman et al.	2018	US	Constraints and facilitators to developing collaborative campus wellness partnerships
55	Jack et al.	2019	UK	Higher Education as a Space for Promoting the Psychosocial Well-being of Refugee Students
Observational study – whole/healthy institution concept is central : data collected specific to this				
56	Sirakamon et al.	2006	Thailand	Policy related to health promotion at Chiang Mai University: administrator views
57	Patterson and Kline	2008	Canada	Report on post-secondary institutions as healthy settings: The pivotal role of student services
58	Dooris and Doherty	2009	UK	National Research and Development Project on Healthy Universities
59	Dooris and Doherty	2010a	UK	Healthy universities-time for action: a qualitative research study exploring the potential for a national programme
60	Dooris and Doherty	2010b	UK	Healthy Universities: current activity and future directions—fìndings and reflections from a nationallevel qualitative research study
61	Newton	2014	UK	Can a university be a ‘healthy university’? An analysis of the concept and an exploration of its operationalisation through two case studies
62	Holt et al.	2015	UK	Student perceptions of a healthy university
63	Newton et al.	2016	UK	Healthy universities: an example of a whole-system health-promoting setting
64	brucks et al.	2017	US	Aligning CSUSM with Healthy Campus 2020: A qualitative needs assessment
65	Sarmiento	2017	US	Healthy universities: mapping health-promotion interventions
66	Dooris et al.	2020	UK	Conceptualising the ‘whole university’ approach: an international qualitative study
Description – actions of a specific institution				
67	bruce	1993	Canada	Implementing a university campus wellness model
68	beattie	1998	UK	Action learning for health on campus: muddling through with a model? University College of St Martin, Lancaster
69	Dooris	1998	UK	The university as a setting for sustainable health
70	Dowding and Thompson	1998	UK	Embracing organizational development for health promotion in higher education: Lancaster University
71	Peterken	1998	UK	The healthy university within a healthy city: University of Portsmouth
72	White	1998	UK	Creating a healthy medical school: University of Newcastle
73	Dooris	2001	UK	The ‘Health Promoting University’: a critical exploration of theory and practice
74	Dooris	2002	UK	The Health Promoting University: opportunities, challenges and future developments
75	Reger et al.	2002	US	Implementing university-based wellness: a participatory planning approach
76	Marshall	2007	UK	bradford College – a healthy college
77	Perlejewski	2007	UK	Yeovil College – our commitment to a better college
78	Vincent	2007	UK	Stoke on Trent College awarded the Kirkless Healthy College Standard
79	Mendenhall et al.	2008	US	Students Against Nicotine and Tobacco Addiction (SANTA): community-based participatory research in a high-risk young adult population
80	Stylianou	2010	UK	‘A Practical Guide to becoming a Healthy College’
81	Mendenhall et al.	2011	US	The SANTA project (Students Against Nicotine and Tobacco Addiction): Using community-based participatory research to reduce smoking in a high-risk young adult population
82	Knight and La Placa	2013	UK	Healthy Universities: taking the University of Greenwich Healthy Universities Inititive forward
83	Harrington	2016	US	‘America’s Healthiest Campus’: The OSU Well-being Strategy Model
84	black	2018	Canada	Designing healthy and supportive campus communities: An example from Simon Fraser University
Evaluation – institution-level: evaluation of intervention in one/small number of institutions NOT formal trial				
85	Xiangyang et al.	2003	China	beijing Health Promoting Universities: practice and evaluation
86	Meier et al.	2007	Germany	The contribution of health discussion groups with students to campus health promotion
87	Budgen et al.	2011	Canada	Creating a healthier campus community using action research and health promotion strategies: Students and organizational leaders as partners
88	Sirakamon et al.	2011	Thailand	Factors influencing the development of a Thai health-promoting faculty of nursing: An ethnographic exploration
89	Mendenhall et al.	2014	US	Community-based participatory research to decrease smoking prevalence in a high-risk young adult population: An evaluation of the Students Against Nicotine and Tobacco Addiction (SANTA) project
90	Sirakamon et al.	2017	Thailand	An ethnography of health-promoting faculty in a Thailand university
Evaluation – larger policy: evaluation of bigger policy intervention across multiple institutions NOT formal trial				
91	burwell et al.	2010	US	Healthy Campus 2010: Midcourse review
92	Dooris and Powell	2012	UK	Developing leadership and governance for Healthy Universities: Final report
93	Dooris et al.	2018	UK	The UK Healthy Universities Self-Review Tool: Whole-system impact
94	Dooris et al.	2019	UK	Whole system approaches in higher education: an evaluation of the UK Healthy Universities Network
95	Suarez-Reyes et al.	2019	belgium	How do universities implement the Health Promoting University concept?
Trial				
96	Reavley et al.	2014a	Australia	A multifaceted intervention to improve mental health literacy in employees of a multicampus university: a cluster randomised trial
97	Reavley et al.	2014b	Australia	A multifaceted intervention to improve mental health literacy in students of a multicampus university: a cluster randomised trial
(Systematic) review: including empirical papers (ie not a review of theoretical papers)				
98	Fernandez et al.	2016	Australia	Setting-based interventions to promote mental health at the university: a systematic review
99	Suarez-Reyes and Van den Broucke	2016	belgium	Implementing the Health Promoting University approach in culturally different contexts: a systematic review
100	Ferreira et al.	2018	Portugal	Health promotion programs in higher education: integrative review of the literature
101	Reis et al.	2018	Portugal	The promotion of Healthy Universities: a systematic review

**Table 2:** Publications with each key term in the title and/or abstract according to publication decade and country (columns may sum to more than total number of publications/more than 100% because all applicable terms within each title/abstract coded).

	DECADE^a^								COUNTRY^b^					
	1971–1980		1991–2000		2001–2010		2011–2020		UK		US		Elsewhere^~^	
	N	(%)	N	(%)	N	(%)	N	(%)	N	(%)	N	(%)	N	(%)
Setting(s)	0	(0)	2	(15)	14	(41)	20	(40)	19	(41)	2	(7)	15	(55)
Any of: System(s)/systemic; whole system; complex system	1	(25)	0	(0)	6	(18)	10	(20)	13	(28)	2	(7)	2	(7)
Participatory action/process/research	0	(0)	0	(0)	2	(6)	4	(8)	0	(0)	4	(14)	2	(7)
Other broad	2	(50)	6	(46)	15	(44)	31	(62)	19	(41)	17	(61)	18	(67)
Healthy(ier) university/college	0	(0)	2	(15)	15	(44)	18	(36)	31	(67)	1	(4)	3	(11)
Health promoting university/college	0	(0)	3	(23)	10	(29)	20	(40)	17	(37)	1	(4)	15	(55)
Healthy(ier) campus	0	(0)	1	(8)	2	(6)	10	(20)	0	(0)	9	(32)	4	(15)
TOTAL PUBLICATIONS PER DECADE/COUNTRY	4		13		34		50		46		28		27	

a % columns = of total publications per decade

b % columns = of total publications per country

~ Canada, Australia, New Zealand, other Europe, Thailand, China

**Table 3:** Number of publications mentioning (1) each group as target of intervention or focus of data-gathering and (2) each health dimension (note lists sum to more than total number of publications as all applicable targets/dimensions within each publication coded).

	N (of 101)
(1) Target of intervention/focus of data-gathering	
Students	87
Staff	73
External/wider community	28
Unclear	1
N/A (data gathered at organisational level)	1
(2) Health dimension	
‘Health’ general/implied	69
Mental health – lower level, wellbeing, self-esteem, confidence, etc.	23
behaviour – nutrition including water	17
behaviour – smoking	17
behaviour – alcohol	16
Mental health – formal psychiatric illness/diagnoses	15
behaviour – physical activity	13
behaviour – sexual	13
behaviour – drugs	12
behaviour – other	8
Use of health services	7
Wholistic health – explicit/dimensions specified	7
Attitudes/knowledge	6
behaviour – violence/aggression/safety	6
Physical health	3

**Table 4:** Where descriptions of interventions available (in descriptions of the activities of individual institutions; evaluations; trial) – who and what was involved?

Who was involved in producing the intervention?	N (max possible = 10)^a^
Students	10
Senior/Managerial staff	9
Health centre staff	8
Other staff	7
Teaching staff	6
Catering/Physical Activity staff	6
External organisations	6
Health promotion staff/team	4
Specific healthy/ health promoting university/college co-ordinator	3
Wider community	1
What activities did the intervention involve?	N (max possible = 14)^b^
Promotions/marketing	14
Health education	13
Policies	9
Survey/data-gathering	9
Health services – changes in what/where/when/to whom provided	8
Staff wellbeing support/counselling services/development opportunities	8
Architecture/greenspace/physical environment	7
Catering – changes in what/where/when/to whom provided	7
Student projects/committees	6
Physical activity – changes in what/where/when/to whom provided	6
Learning – curriculum/classroom environment/exams/etc	6
‘Partnerships’/’Relationship-building’/communication (styles)	5
Staff training	4
Student peer-to-peer (education/support/counselling/relationships)	3
Dedicated website	2
Procurement	2
Other	5

a Of the 19, four were unclear but comments suggested wide involvement and five were unclear and provided no clear comments on involvement.

b Of the 19, three were unclear but comments suggested wide-ranging activities and two were unclear and provided no clear comments on activities

## Data Availability

Data sharing is not applicable to this article as no new data were created or analysed in this study.

## References

[R1] Ahern P (2007). Stockport Healthy College Standard: An Audit Tool for Every Child Matters. Education & Health.

[R2] American College Health Association (2018). Healthy Campus.

[R3] Arksey H, O’Malley L (2005). Scoping Studies: Towards a Methodological Framework. International Journal of Social Research Methodology.

[R4] Balding A (2007). Support for Healthy Colleges. Education & Health.

[R5] Beattie A, Tsouros AD, Dowding G, Thompson J, Dooris M (1998). Health Promoting Universities: Concept, Experience and Framework for Action.

[R6] Bennett LM, Gadlin H (2012). Collaboration and Team Science: From Theory to Practice. Journal of Investigative Medicine.

[R7] Birch K (2006). Kirklees Healthy College Standard. Education and Health.

[R8] Black T, Cassidy W, Faucher C, Jackson M (2018). Cyberbullying at University in International Contexts.

[R9] Broglia E, Millings A, Barkham M (2018). Challenges to Addressing Student Mental Health in Embedded Counselling Services: A Survey of Uk Higher and Further Education Institutions. British Journal of Guidance & Counselling.

[R10] Bruce G (1993). Implementing a University Campus Wellness Model. American Association of Occupational Health Nurses Journal.

[R11] Brucks L, Majid S, Parlin N (2017). Aligning CSUSM with Healthy Campus 2020: A Qualitative Needs Assessment.

[R12] Budgen C, Fedderson M, Sullivan K, Cull I, Mchugh N, Fedderson M, Sullivan K (2011). Creating a Healthier Campus Community Using Action Research and Health Promotion Strategies: Students and Organizational Leaders as Partners. International Journal of Health, Wellness and Society.

[R13] Burwell C, Dewald L, Grizzell J (2010). Healthy Campus 2010: Midcourse Review. American Journal of Health Studies.

[R14] Came H, Tudor K (2020). The Whole and Inclusive University: A Critical Review of Health Promoting Universities from Aotearoa New Zealand. Health Promotion International.

[R15] Canadian Mental Health Association (2020). Healthy Minds, Healthy Campuses.

[R16] Canadian Mental Health Association (2013). Post-secondary Student Mental Health: Guide to a Systemic Approach.

[R17] Capewell S, Capewell A (2017). An Effectiveness Hierarchy of Preventive Interventions: Neglected Paradigm or Self-evident Truth?. Journal of Public Health.

[R18] Cooke A, Smith D, Booth A (2012). Beyond PICO: The SPIDER Tool for Qualitative Evidence Synthesis. Qualitative Health Research.

[R19] Craig P, Dieppe P, Macintyre S, Michie S, Nazareth I, Petticrew M (2008). Developing and Evaluating Complex Interventions: The New Medical Research Council Guidance. British Medical Journal.

[R20] Doherty S, Cawood J, Dooris M (2011). Applying the Whole-system Settings Approach to Food within Universities. Perspectives in Public Health.

[R21] Doherty S, Dooris M (2006). The Healthy Settings Approach: The Growing Interest within Colleges and Universities. Education and Health.

[R22] Dooris M, Tsouros AD, Dowding G, Thompson J, Dooris M (1998). Health Promoting Universities: Concept, Experience and Framework for Action.

[R23] Dooris M (2001). The ‘Health Promoting University’: A Critical Exploration of Theory and Practice. Health Education.

[R24] Dooris M (2002). The Health Promoting University: Opportunities, Challenges and Future Developments. Promotion & Education.

[R25] Dooris M (2006). Healthy Settings: Challenges to Generating Evidence of Effectiveness. Health Promotion International.

[R26] Dooris M, Poland B, Kolbe L, De Leeuw E, Mccall D, Wharf-Higgins J, Mcqueen D, Jones C (2007). Global Perspectives on Health Promotion Effectivness.

[R27] Dooris M (2010). Healthy Universities: Introduction and Model.

[R28] Dooris M (2013). Expert Voices for Change: Bridging the Silos - Towards Healthy and Sustainable Settings for the 21st Century. Health & Place.

[R29] Dooris M, Doherty S, Orme J, Mittelmark MB, Sagy S, Eriksson M, Bauer GF, Pelikan JM, Lindström B, E G (2017). The Handbook of Salutogenesis.

[R30] Dooris M, Farrier A, Doherty S, Holt M, Monk R, Powell S (2018). The UK Healthy Universities Self-review Tool: Whole-system Impact. Health Promotion International.

[R31] Dooris M, Farrier A, Powell S, Holt M (2019). Whole System Approaches in Higher Education: An Evaluation of the UK Healthy Universities Network. Health Education.

[R32] Dooris M, Cawood J, Doherty S, Powell S (2010). Healthy Universities: Concept, Model and Framework for Applying the Healthy Settings Approach within Higher Education in England.

[R33] Dooris M, Wills J, Newton J (2014). Theorizing Healthy Settings: A Critical Discussion with Reference to Healthy Universities. Scandinavian Journal of Public Health.

[R34] Dooris M, Doherty S (2008). English Healthy Universities Network: Framework for Action.

[R35] Dooris M, Doherty S (2009). National Research and Development Project on Healthy Universities.

[R36] Dooris M, Doherty S (2010a). Healthy Universities – time for Action: A Qualitative Research Study Exploring the Potential for A National Programme. Health Promotion International.

[R37] Dooris M, Doherty S (2010b). Healthy Universities: Current Activity and Future Directions – findings and Reflections from a National-level Qualitative Research Study. Global Health Promotion.

[R38] Dooris M, Powell S (2012). Developing Leadership and Governance for Healthy Universities: Final Report.

[R39] Dooris M, Powell S, Farrier A (2020). Conceptualizing the ‘Whole University’ Approach: An International Qualitative Study. Health Promotion International.

[R40] Dowding G, Thompson J, Tsouros AD, Dowding G, Thompson J, Dooris M (1998). Health Promoting Universities: Concept, Experience and Framework for Action.

[R41] Drum DJ, Denmark Ab (2012). Campus Suicide Prevention: bridging Paradigms and Forging Partnerships. Harvard Review of Psychiatry.

[R42] Fabiano P, Swinford PL (2004). Serving Higher Education Communities with Health Promotion. American Journal of Health Promotion.

[R43] Fernandez A, Howse E, Rubio-Valera M, Thorncraft K, Noone J, Luu X, Veness B, Leech M, Llewellyn G, Salvador-Carulla L (2016). Setting-based Interventions to Promote Mental Health at the University: A Systematic Review. International Journal of Public Health.

[R44] Ferreira FMPB, Brito IS, Santos MR (2018). Health Promotion Programs in Higher Education: Integrative Review of the Literature. Revista Brasileira De Enfermagem.

[R45] Filkowski MB (2008). Leadership for Campus Mental Wellness.

[R46] Frieden T (2010). A Framework for Public Health Action: The Health Impact Pyramid. American Journal of Public Health.

[R47] Gale J, Thalitaya M (2015). Mental Health Support Service for University Students. Psychiatria Danubina.

[R48] Glazer WM (1979). General Systems Theory and College Mental Health Professionals. Journal of American College Health Association.

[R49] Gordon K (1995). College Health in the National blueprint for a Healthy Campus 2000. Journal of American College Health.

[R50] Haas J, Baber M, Byrom N, Meade L, Nouri-Aria K (2018). Changes in Student Physical Health behaviour: An Opportunity to Turn the Concept of a Healthy University into a Reality. Perspectives in Public Health.

[R51] Harrington S (2016). “America’s Healthiest Campus”: The OSU Well-being Strategy Model. American Journal of Health Promotion.

[R52] Hartman CL, Evans KE, Barcelona RJ, Brookover RS (2018). Constraints and Facilitators to Developing Collaborative Campus Wellness Partnerships. Recreational Sports Journal.

[R53] Hewitt K (1976). The Whole College Catalog about Drinking: A Guide to Alcohol Abuse Prevention.

[R54] Hoffman T, Glasziou P, Boutron I, Milne R, Perera R, Moher D, Altman D (2014). Better Reporting of Interventions: Template for Intervention Description and Replication (TIDieR) Checklist and Guide. BMJ.

[R55] Holt M, Monk R, Powell S, Dooris M (2015). Student Perceptions of a Healthy University. Public Health.

[R56] Holt M, Powell S (2017). Healthy Universities: A Guiding Framework for Universities to Examine the Distinctive Health Needs of Its Own Student Population. Perspectives in Public Health.

[R57] Horacek T, Simon M, Yildirim E, White A, Shelnutt K, Riggsbee K, Olfert M (2019). Development and Validation of the Policies, Opportunities, Initiatives and Notable Topics (POINTS) Audit for Campuses and Worksites. International Journal of Environmental Research and Public Health.

[R58] Innstrand S, Christensen M (2020). Healthy Universities. The Development and Implementation of a Holistic Health Promotion Intervention Programme Especially Adapted for Staff Working in the Higher Educational Sector: The ARK Study. Global Health Promotion.

[R59] (2015). Okanagan Charter: An International Charter for Health Promoting Universities and Colleges.

[R60] Jack O, Chase E, Warwick I (2019). Higher Education as a Space for Promoting the Psychosocial Well-being of Refugee Students. Health Education Journal.

[R61] Jackson ML, Weinstein HM (1997). The Importance of Healthy Communities of Higher Education. Journal of American College Health.

[R62] James K (2003). A Health Promoting College for 16-19 Year Old Learners.

[R63] Keshavarz N, Nutbeam D, Rowling L, Khavarpour F (2010). Schools as Social Complex Adaptive Systems: A New Way to Understand the Challenges of Introducing the Health Promoting Schools Concept. Social Science & Medicine.

[R64] Knight A, La Placa V (2013). Healthy Universities: Taking the University of Greenwich Healthy Universities Inititive Forward. International Journal of Health Promotion and Education.

[R65] Langford R, Bonell C, Jones H, Pouliou T, Murphy S, Waters E, Komro K, Gibbs L, Magnus D, Campbell R (2015). The World Health Organization’s Health Promoting Schools Framework: A Cochrane Systematic Review and Meta-analysis. BMC Public Health.

[R66] Lederer AM, Oswalt SB (2017). The Value of College Health Promotion: A Critical Population and Setting for Improving the Public’s Health. American Journal of Health Education.

[R67] Lewis MA, Fitzgerald TM, Zulkiewicz B, Peinado S, Williams PA (2017). Identifying Synergies in Multilevel Interventions: The Convergence Strategy. Health Education & behavior.

[R68] Liu C, Pinder-Amaker S, Hahm H, Chen J (2020). Priorities for Addressing the Impact of the COVID-19 Pandemic on College Student Mental Health. Journal of American College Health.

[R69] Marshall J (2007). Bradford College - a Healthy College. Education & Health.

[R70] Marshall J, Stylianou H (2010). A Practical Guide to becoming A Healthy College.

[R71] Mays N, Roberts E, Popay J, Fulop N, Allen P, Clark A, Black N (2001). Studying the Organization and Delivery of Health Services: Research Methods.

[R72] Mcleroy KR, Bibeau D, Steckler A, Glanz K (1988). An Ecological Perspective on Health Promotion Programs. Health Education Quarterly.

[R73] Meier S, Stock C, Krämer A (2007). The Contribution of Health Discussion Groups with Students to Campus Health Promotion. Health Promotion International.

[R74] Mendenhall T, Whipple H, Harper P, Haas S (2008). Students against Nicotine and Tobacco Addiction (SANTA): Community-based Participatory Research in a High-risk Young Adult Population. Families, Systems and Health.

[R75] Mendenhall T, Harper P, Stephenson H, Santo Haas G (2011). The SANTA Project (Students against Nicotine and Tobacco Addiction): Using Community-based Participatory Research to Reduce Smoking in a High-risk Young Adult Population. Action Research.

[R76] Mendenhall T, Harper PG, Henn L, Rudser KD, Schoeller BP (2014). Communitybased Participatory Research to Decrease Smoking Prevalence in a High-risk Young Adult Population: An Evaluation of the Students against Nicotine and Tobacco Addiction (SANTA) Project. Families, Systems, & Health.

[R77] Murphy E (2017). Responding to the Needs of Students with Mental Health Difficulties in Higher Education: An Irish Perspective. European Journal of Special Needs Education.

[R78] National Association of Student Personnel Administrators (NASPA) (2004). Leadership for a Healthy Campus: An Ecological Approach for Student Success.

[R79] National Institute on Alcohol Abuse and Alcoholism (2019). Planning Alcohol Interventions Using NIAAA’s CollegeAIM’ Alcohol Intervention Matrix.

[R80] Newton J (2014). Can a University Be a ‘Healthy University’? An Analysis of the Concept and an Exploration of Its Operationalisation through Two Case Studies.

[R81] Newton J, Dooris M, Wills J (2016). Healthy Universities: An Example of a Whole-system Health-promoting Setting. Global Health Promotion.

[R82] O’Cathain A, Croot L, Duncan E, Rousseau N, Sworn K, Turner KM, Yardley L, Hoddinott P (2019). Guidance on How to Develop Complex Interventions to Improve Health and Healthcare. BMJ Open.

[R83] O’Donnell T, Gray G (1993). The Health Promoting College.

[R84] Orme J, Dooris M (2010). Integrating Health and Sustainability: The Higher Education Sector as a Timely Catalyst. Health Education Research.

[R85] Partnership for a Healthier America Healthier Campus Initiative.

[R86] Patrick K, Grace TW, Lovato CY (1992). Health Issues for College Students. Annual Review of Public Health.

[R87] Patterson P, Kline T (2008). Report on Post-secondary Institutions as Healthy Settings: The Pivotal Role of Student Services.

[R88] Perlejewski A (2007). Yeovil College - Our Commitment to a Better College. Education and Health.

[R89] Peterken C, Tsouros AD, Dowding G, Thompson J, Dooris M (1998). Health Promoting Universities: Concept, Experience and Framework for Action.

[R90] Racher FE, Hyndman K, Anonson J, Arries E, Foster C (2014). Taking the Right Action in the Right Way: A Comparison of Frameworks for Assessing the Health and Quality of Life of A Postsecondary Student Campus Community. Research and Theory for Nursing Practice.

[R91] Reavley NJ, McCann TV, Cvetkovski S, Jorm AF (2014a). A Multifaceted Intervention to Improve Mental Health Literacy in Employees of A Multicampus University: A Cluster Randomised Trial. Journal of Public Mental Health.

[R92] Reavley NJ, McCann TV, Cvetkovski S, Jorm AF (2014b). A Multifaceted Intervention to Improve Mental Health Literacy in Students of A Multicampus University: A ClusterRandomised Trial. Social Psychiatry and Psychiatric Epidemiology.

[R93] Reger B, Williams K, Kolar M, Smith H, Douglas J (2002). Implementing University-based Wellness: A Participatory Planning Approach. Health Promotion Practice.

[R94] Reis M, Ramiro L, Gomez-Baya D, De Matos MG (2018). The Promotion of Healthy Universities: A Systematic Review. CPQ Women and Child Health.

[R95] Rogers PJ (2008). Using Programme Theory to Evaluate Complicated and Complex Aspects of Interventions. Evaluation.

[R96] Sarmiento JP (2017). Healthy Universities: Mapping Health-promotion Interventions. Health Education.

[R97] (2006). The Edmonton Charter for Health Promoting Universities and Institutions of Higher Education.

[R98] Shah M, Nair CS, Richardson JTE (2017). Measuring and Enhancing the Student Experience.

[R99] Shareck M, Frohlich K, Poland B (2013). Reducing Social Inequities in Health through Settings-related Interventions - a Conceptual Framework. Global Health Promotion.

[R100] Sirakamon S, Chontawan R, Akkadechanun T, Turale S (2011). Factors Influencing the Development of a Thai Health-promoting Faculty of Nursing: An Ethnographic Exploration. Nursing & Health Sciences.

[R101] Sirakamon S, Chontawan R, Akkadechanun T, Turale S (2017). An Ethnography of Health-promoting Faculty in a Thailand University. Health Promotion International.

[R102] Sirakamon S, Kunaviktikul W, Chontawan R, Skillen DL (2006). Policy Related to Health Promotion at Chiang Mai University: Administrator Views. Chiang Mai Medical Bulletin.

[R103] Sowers MF, Colby S, Greene GW, Pickett M, Franzen-Castle L, Olfert MD, Shelnutt K, Brown O, Horacek TM, Kidd T (2017). Survey Development to Assess College Students’ Perceptions of the Campus Environment. American Journal of Health Behavior.

[R104] Stock C, Mcalaney J, Pischke C, Vriesacker B, Van Hal G, Akvardar Y, Orosova O, Kalina O, Guillen-Grima F, Bewick BM (2014). Student Estimations of Peer Alcohol Consumption: Links between the Social Norms Approach and the Health Promoting University Concept. Scandinavian Journal of Public Health.

[R105] Stylianou H (2010). A Practical Guide to becoming A Healthy College. Education & Health.

[R106] Suarez-Reyes M, Serrano M, Van Den Broucke S (2019). How Do Universities Implement the Health Promoting University Concept?. Health Promotion International.

[R107] Suarez-Reyes M, Van Den Broucke S (2016). Implementing the Health Promoting University Approach in Culturally Different Contexts: A Systematic Review. Global Health Promotion.

[R108] Taylor P, Saheb R, Howse E (2017). Creating Healthier Graduates, Campuses and Communities: Why Australia Needs to Invest in Health Promoting Universities. Health Promotion Journal of Australia.

[R109] Thomas F, Aggleton P (2016). A Confluence of Evidence: What Lies behind A “Whole School” Approach to Health Education in Schools?. Health Education.

[R110] Torp S, Vinje HF (2014). Is Workplace Health Promotion Research in the Nordic Countries Really on the Right Track?. Scandinavian Journal of Public Health.

[R111] Tsouros AD, Dowding G, Tsouros AD, Dowding G, Thompson J, Dooris M (1998). Health Promoting Universities: Concept, Experience and Framework for Action.

[R112] Tsouros AD, Tsouros AD, Dowding G, Thompson J, Dooris M (1998). Health Promoting Universities; Concept, Experience and Framework for Action.

[R113] Tsouros AD, Dowding G, Dooris M, Tsouros AD, Dowding G, Thompson J, Dooris M (1998). Health Promoting Universities: Concept, Experience and Framework for Action.

[R114] University of Central Lancashire (2015). UCLan Healthy University Action Plan 2015-18.

[R115] University of Central Lancashire & Manchester Metropolitan University Healthy Universities.

[R116] Vincent D (2007). Stoke on Trent College awarded the Kirklees Healthy College Standard. Education & Health.

[R117] Warwick I, Maxwell C, Simon A, Statham J, Aggleton P (2006). Mental Health and Emotional Well-being of Students in Further Education - a Scoping Study.

[R118] Warwick I, Statham J, Aggleton P (2008). Healthy and Health Promoting Colleges - Identifying an Evidence base.

[R119] Western Interstate Commission for Higher Education (1973). The Ecosystem Model: Designing Campus Environments.

[R120] White M, Tsouros AD, Dowding G, Thompson J, Dooris M (1998). Health Promoting Universities: Concept, Experience and Framework for Action.

[R121] Whitehead D (2004). The Health Promoting University (HPU): The Role and Function of Nursing. Nurse Education Today.

[R122] Whitelaw S, Baxendale A, Bryce C, Machardy L, Young I, Witney E (2001). ‘Settings’ based Health Promotion: A Review. Health Promotion International.

[R123] Winer JA, Dinello FA, Pasca A, Weingarten S (1974). Innovations at University Mental Health Services. Journal of the American College Health Association.

[R124] (1986). World Health Organisation.

[R125] World Health Organisation (2018). Global Standards for Health Promoting Schools.

[R126] Xiangyang T, Lan Z, Xueping M, Tao Z, Yuzhen S, Jagusztyn M (2003). Beijing Health Promoting Universities: Practice and Evaluation. Health Promotion International.

[R127] Zimmer CG, Hill MH, Sonnad SR (2003). A Scope-of-practice Survey Leading to the Development of Standards of Practice for Health Promotion in Higher Education. Journal of American College Health.

